# Supplementary material for: Natural Allelic Variations of Bch10G006400 Controlling Seed Size in Chieh-qua (Benincasa hispida Cogn. var. Chieh-qua How)
Source: Int J Mol Sci. 2024 Apr 11;25(8):4236. doi: 10.3390/ijms25084236 (PMC11050567; doi:10.3390/ijms25084236)
Supplement: Supplementary file 1 [file ijms-25-04236-s001.zip › ijms-2897532-supplementary.pdf]

Title: Natural allelic variations of *Bch10G006400* controlling seed size in chieh-qua (*Benincasa hispida* Cogn. var. *Chieh-qua* How).

Supplementary Information

Table S1. Primer use for linkage analysis of seed size.

Primer Name	Position	Type	Forward primer (5'→3')	Reverse primer (5'→3')
**SS_1**	15,351,120	Indel	GAGAGTAAAAAGTTCTAAATATCCG	CTATCTCCACTGTACTAGAAAAATC
**SS_2**	15,867,600	Indel	ACATCACACTACACGTTCGTATTGG	TCAAAACTCACCATCAGCTCTACAA
**SS_3**	15,889,360	Indel	CACTGTTTATAGGCAAAATGTCAAG	ACAACCTATAAGTTTAACCCCATGT
**SS_4**	15,938,160	Indel	GTCTATGTAATCCACCTCTCACTTG	AAGGAGTACACGATGTACAACAAGA
**SS_5**	15,964,480	Indel	CACTACATTACTCATTTGGGAGTAG	CAGAGGCTACTATTGCTCACTCTAT
**SS_6**	16,024,640	Indel	GTGTAACTTACGTAATTTCTAACCA	GAAGTCTCAATTTTACGCATATTAC
**SS_7**	16,836,320	Indel	AAAAAAAAGAGGGGCCTATATACAA	TAATCTGCTGTTCTTCAAAACCAAT
**SS_8**	15,906,480	Indel	CTTTTTGTACGTGGATATATCTGGA	TAGACACGGCGAATAACAACTAATA
**SS_9**	15981440	Indel	CAAATATATAAGTTCCAACTCACGC	TAATGTCAACTCATTATGAACCACG
**SS_10**	15988960	Indel	CGAAATGATTAGCAAAAGAGCCTTA	CAAAATAAAATGTACAAGCAGTGGG
**SS_11**	15,997,840	Indel	GCGGAATTTTCATAGTGATTTTGAG	GCCCTCTCTCTCTATCTTTTTTTTC

Table S2. Primers for qRT-PCR and gene cloning.

	Name	Forward primer (5'→3')	Reverse primer (5'→3')
**Gene cloning**	*Bch10G006400*_CDs1	GGACAGTTGGACAGGTATCG	TGGGGAAGAACTCTGTTGAG
	*Bch10G006400*_CDs2	AGACCCAGTAAGAAATCGTC	CTGGTCGTACAAAACATCG
	*Bch10G006400*_sanger1	GACAAGGAACACGTTCTATCA	TCTCCCACTCTTCCAACTC
	*Bch10G006400*_sanger2	ATTACGAGTTGGAAGAGTGG	CTCGTTGTAATCTGCCTGT
	*Bch10G006400*_sanger3	TCAAACAACCGCCATG	GAACAAACCCTAGTTAAATCC
**Primers of qRT-PCR**	*Bch10G006400*_qPCR	AAACAGGCAGATTACAACG	ATCAGGCACCAAAACAAG
	Actin-UBQ	CCTTGCCGACTACAACATCC	GCACTCCTTCCTAAGCCTCT

Table S3. Seed size linkage markers.

**314SNP-dCAPS**	**Locus sequence**	J16:TTCTTGGCTTGAGAGTGGCGCCGCCGTATCGCCGCCGTGGGATTGG<br>FJ5:TTCTTGGCTTGAGAGTGGCGCCGCCGTATCCCCGCCGTGGGATTGG
	**Forward primer (5'→3')**	TTCTTGGCTTGAGAGTGGCGCCGCCGGATC
	**Reverse primer (5'→3')**	GCATGATTATCTTTCTCAGTGGC
	**Endonuclease**	*BamHI*
	**Recognition site**	GGATCC
**400SNP-dCAPS**	**Locus sequence**	J16:GTTGATTACTGTTGCATGGCCACTGAGAAAGATAATCATGCCTCTCTTA
		FJ5:GTTGATTACTGTTGCATGGCCACTGAGAAAGATAATCATGCCTCTCTTA
		17097:GTTGATTACTGTTGCATGGCCACTTAGAAAGATAATCATGCCTCTCTTA
	**Forward primer (5'→3')**	GTTGATTACTGTTGCATGGCCTCT
	**Reverse primer (5'→3')**	CTGATGAATTGATATTGTATGGACG
	**Endonuclease**	*Hpy188I*
	**Recognition site**	TCNGA
**551_InDel**	**Locus sequence**	J16:CAACATTCAAAAGCTAAAAATAGAAGAAGCAGAAGCAATATAC
		FJ5:CAACATTCAAAAGCTAAAAATAGAAGAAGCAGAAGCAATATACAA
		1714:CAACATTCAAAAGCTAAAAAT---AGAAGCAGAAGCAATATACAA

	Reverse primer (5'→3')	TAGTAGACCCAGTAAGAAATCG

Note: Tag name prefixes 314, 400, and 551 represent coded location points, red bold represents mismatch, green bold represents variation sites, and - represents deletion.

Table S4. Genotyping results of 108 germplasm chieh-qua materials.

ID	Sample name	SNP _314	SNP _400	InDel _551	Phenotype	ID	Sample name	SNP _314	SNP _400	InDel_ 551	Phenotype
1	0401	**G**	G	AAG	Large seed	55	1732	G	**T**	AAG	Small seed
2	0504	**G**	G	AAG	Large seed	56	1733	G	**T**	AAG	Small seed
3	A-2	**G**	G	AAG	Large seed	57	17046	G	**T**	AAG	Small seed
4	B-4	**G**	G	AAG	Large seed	58	17048	G	**T**	AAG	Small seed
5	C-3	**G**	G	AAG	Large seed	59	17050	G	**T**	AAG	Small seed
6	D2	**G**	G	AAG	Large seed	60	17051	G	**T**	AAG	Small seed
7	D21-4	**G**	G	AAG	Large seed	61	SD19	G	**T**	AAG	Small seed
8	D3	**G**	G	AAG	Large seed	62	17097	G	**T**	AAG	Small seed
9	D4	**G**	G	AAG	Large seed	63	18FJ	G	**T**	AAG	Small seed
10	D4-1	**G**	G	AAG	Large seed	64	17010	G	**T**	AAG	Small seed
11	D7	**G**	G	AAG	Large seed	65	17023	G	**T**	AAG	Small seed
12	D10	**G**	G	AAG	Large seed	66	17057	G	**T**	AAG	Small seed
13	D16	**G**	G	AAG	Large seed	67	FJ1022	G	**T**	AAG	Small seed
14	D19-6	**G**	G	AAG	Large seed	68	18FJ-2	G	**T**	AAG	Small seed
15	D19-20	**G**	G	AAG	Large seed	69	18FJ14	G	**T**	AAG	Small seed
16	D20-7	**G**	G	AAG	Large seed	70	17087	G	**T**	AAG	Small seed
17	F-2	**G**	G	AAG	Large seed	71	17016	G	**T**	AAG	Small seed
18	J1745	**G**	G	AAG	Large seed	72	17067	G	**T**	AAG	Small seed
19	FJ3	**G**	G	AAG	Large seed	73	1730	G	G	-	Small seed
20	Group A	**G**	G	AAG	Large seed	74	17081	G	G	-	Small seed
21	Tai 13	**G**	G	AAG	Large seed	75	1718	G	G	-	Small seed
22	J25	**G**	G	AAG	Large seed	76	17055	G	G	-	Small seed
23	D22-5	**G**	G	AAG	Large seed	77	17058	G	G	-	Small seed
24	D22-3	**G**	G	AAG	Large seed	78	22-2	G	G	-	Small seed
25	D14	**G**	G	AAG	Large seed	79	19FJ4-1	G	G	-	Small seed
26	D22-1	**G**	G	AAG	Large seed	80	19FJ12	G	G	-	Small seed
27	D25-2-1	**G**	G	AAG	Large seed	81	17083	G	G	-	Small seed
28	D21-5	**G**	G	AAG	Large seed	82	0404	G	G	-	Small seed
29	1517	**C**	G	AAG	Small seed	83	17076	G	G	-	Small seed
30	FD1680	**C**	G	AAG	Small seed	84	18FJ4	G	G	-	Small seed
31	17029	**C**	G	AAG	Small seed	85	17002	G	G	-	Small seed
32	17075	**C**	G	AAG	Small seed	86	17069	G	G	-	Small seed
33	17094	**C**	G	AAG	Small seed	87	1722	G	G	-	Small seed
34	1735	**C**	G	AAG	Small seed	88	1709	G	G	-	Small seed
35	17044	**C**	G	AAG	Small seed	89	18FJ18	G	G	-	Small seed
36	17063	**C**	G	AAG	Small seed	90	17080	G	G	-	Small seed
37	17011	**C**	G	AAG	Small seed	91	17054	G	G	-	Small seed

38	17071	C	G	AAG	Small seed	92	17038	G	G	-	Small seed
39	18FJ17	C	G	AAG	Small seed	93	17062	G	G	-	Small seed
40	17041	C	G	AAG	Small seed	94	1749	G	G	-	Small seed
41	17060	C	G	AAG	Small seed	95	17079	G	G	-	Small seed
42	17059	C	G	AAG	Small seed	96	17053	G	G	-	Small seed
43	19FJ2	C	G	AAG	Small seed	97	17061	G	G	-	Small seed
44	19FJ14-2	C	G	AAG	Small seed	98	18FJ19	G	G	-	Small seed
45	17032	C	G	AAG	Small seed	99	PJI-3-1	G	G	-	Small seed
46	17031	C	G	AAG	Small seed	100	17091	G	G	-	Small seed
47	17064	C	G	AAG	Small seed	101	17096	G	G	-	Small seed
48	17093	C	G	AAG	Small seed	102	17045	G	G	-	Small seed
49	19FJ5	C	G	AAG	Small seed	103	1737-1-1	G	G	-	Small seed
50	19FJ26	C	G	AAG	Small seed	104	1729-1	G	G	-	Small seed
51	17085	C	G	AAG	Small seed	105	1714	G	G	-	Small seed
52	1708-2	C	G	AAG	Small seed	106	17084	G	G	-	Small seed
53	19FJ1-1	G	T	AAG	Small seed	107	19FJ28-2	G	G	-	Small seed
54	19FJ9	G	T	AAG	Small seed	108	17070	G	G	-	Small seed

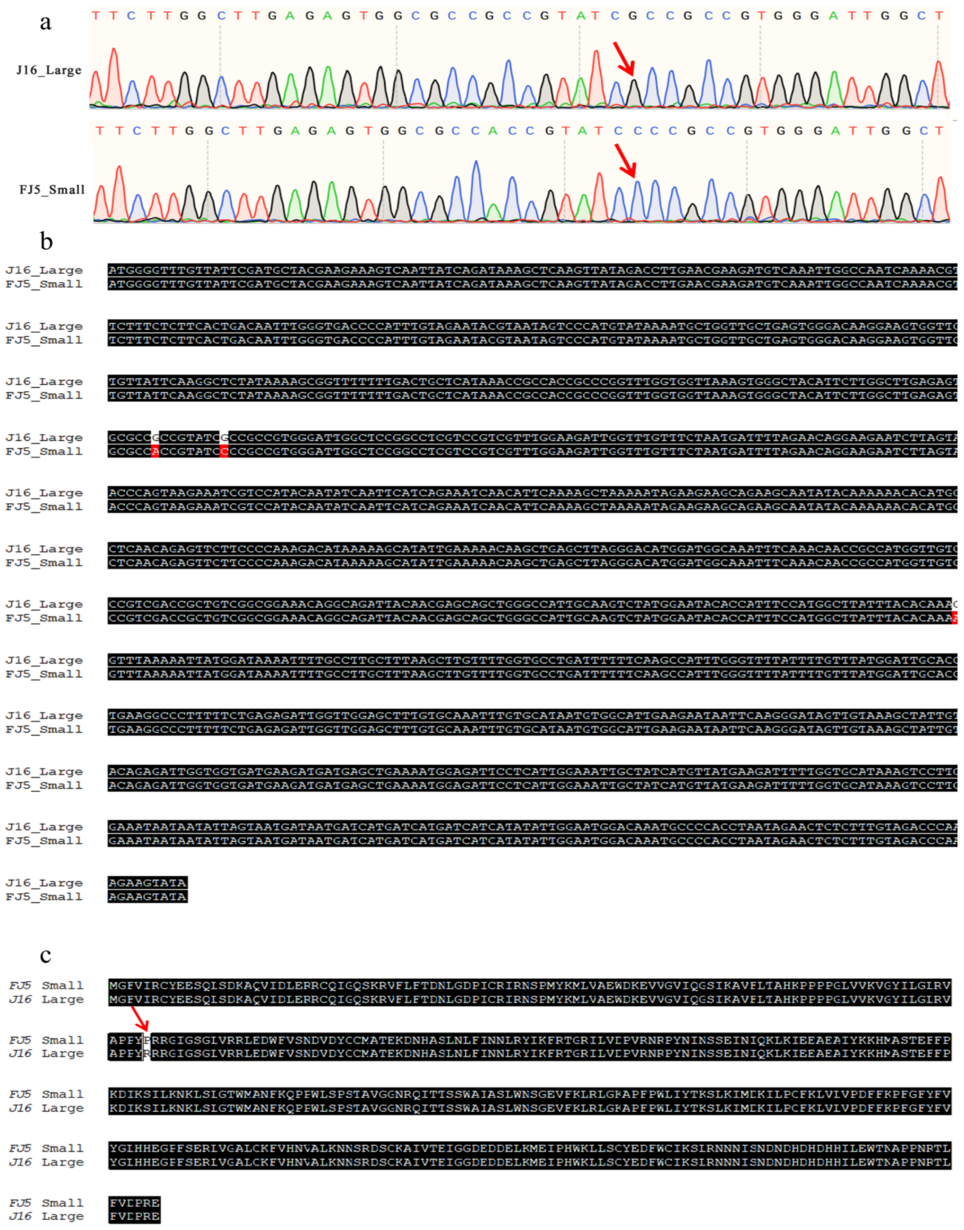

Figure S1. *Bch10G006400* CDS sequence and amino acid comparison in both parents. (**a**) Parental sequencing peak map. (**b**) Parental CDS sequence alignment. (**c**) Parental amino acid sequence alignment.

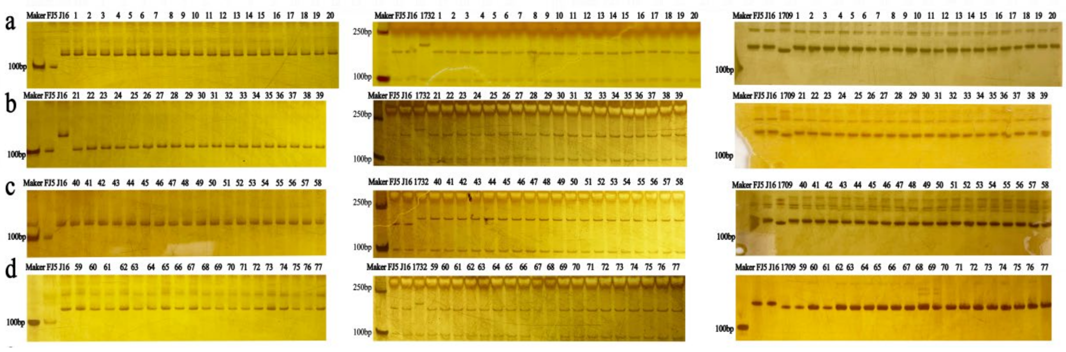

Figure S2. Typing results of 3 markers in some materials. (**a**) The classification results of 20 large materials (G-G-AGA). (**b**) (**c**) (**d**) were the typing results of three haplotypes of small seed materials (C-G-AGA, G-T-AGA and G-G), respectively.

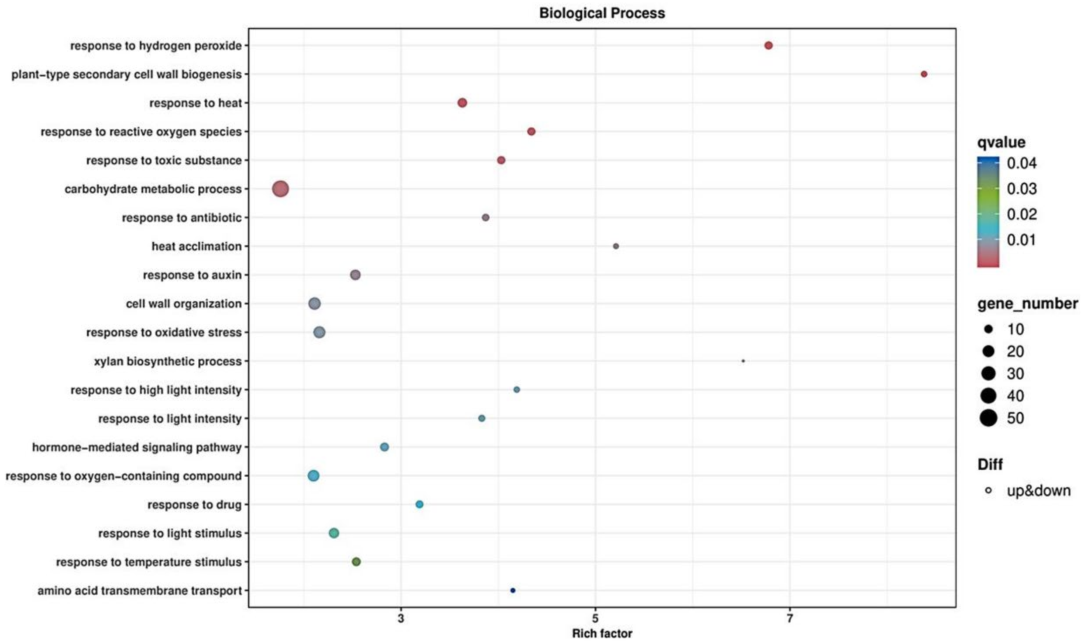

Figure S3. GO pathway enrichment on GOs-Bubble chart. **Note:** The horizontal coordinate is GeneRatio, that is, the proportion of the genes of interest in this entry to the number of differentially expressed genes, and the vertical coordinate is each GO comment entry. The size of the dots represents the number of differentially expressed genes annotated in the pathway, and the color of the dots represents the q value of the hypergeometric test.
